# Qualitative and quantitative evaluation of a standardized training model for improving patients' ability to use inhalers

**DOI:** 10.3389/fpubh.2023.1065311

**Published:** 2023-04-17

**Authors:** Yuwen Huang, Fangzhou Miao, Yunjian Dai, Cuie Chang, Xiaoyu Zhang, Haibin Dai

**Affiliations:** ^1^Department of Pharmacy, Second Affiliated Hospital, Zhejiang University School of Medicine, Hangzhou, China; ^2^Department of Pharmacy, Peking University Third Hospital, Beijing, China

**Keywords:** inhaler, inhalation technique, training, asthma, qualitative evaluation, quantitative evaluation

## Abstract

**Objective:**

Training contributes to the effectiveness of aerosol inhalation therapy. However, qualitative and quantitative evaluation of effective training methods is rarely reported. This study aimed to evaluate the effectiveness of a standardized training model by pharmacists based on verbal instruction and physical demonstration in improving patients' ability to use inhalers using qualitative and quantitative methods. Risk or protective factors affecting correct inhaler use were also explored.

**Methods:**

431 Outpatients with asthma or COPD were recruited and randomly divided into a standardized training group (*n* = 280) and a usual training group (control group, *n* = 151). A framework of qualitative (e.g., multi-criteria analysis) and quantitative comparisons [percentage of correct use (CU%), percentage of complete error (CE%), and percentage of partial error (PE%)] was established to evaluate the two training models. In addition, the changes of key factors (age, education level, adherence, device type, etc.) influencing patients' ability to use inhalers of two models were observed.

**Results:**

The multi-criteria analysis showed that the standardized training model had comprehensive advantages in qualitative indicators. The average correct use percentage (CU%) of the standardized training group was significantly higher than that of the usual training group (77.6% vs. 35.5%). A stratified analysis further demonstrated that the ORs (95%CI) in the usual training group of age and educational level was 2.263 (1.165–4.398) and 0.556 (0.379–0.815), while in the standardized training group, age and educational level were not the key factors influencing the ability to use inhaler devices (*P* > 0.05). Logistic regression analysis demonstrated that standardized training was a protective factor for inhalation ability.

**Conclusion:**

These findings indicate that the framework of qualitative and quantitative comparisons could be used to evaluate training models, and the standardized training model by pharmacists can significantly improve patients' ability to use inhalers correctly and address the influence of older age and lower education because of its methodological advantages. Further studies with more extended follow-up are needed to validate the role of the standardized training model by pharmacists in the correct use of inhalers.

**Clinical trial registration:**

chictr.org.cn, ChiCTR2100043592 (23-02-2021).

## 1. Introduction

Inhaled therapeutic drugs are important in treating asthma and chronic obstructive pulmonary disease (COPD). Inhalers are the most common medication device used in asthma or COPD therapy. The primary advantage of inhaled aerosol treatment involving inhaled medications and their corresponding inhalers is to directly treat the lungs in smaller doses, with fewer side effects than oral delivery (1). However, the effectiveness of inhaled medications is limited by the patient's ability to use the inhaler correctly, and the issue is often neglected when these medications are prescribed (2). Incorrect use of inhalers by patients may lead to uncontrolled asthma or COPD and increased costs (3). There is evidence that costs increase significantly with the severity of asthma (4), and patients with uncontrolled asthma spend more than twice as much on health care as patients with controlled asthma (5). Many international organizations and academic groups, including the International Primary Care Respiratory Group (IPCRG), Aerosol Drug Management Improvement Team (ADMIT), and American Association of Respiratory Care (AARC), are calling for greater awareness that proper use of devices is the key to successful treatment, and have developed corresponding guidelines for implementing them (2, 6–11).

Incorrect inhaler usage may directly result from poor instruction and can be significantly improved by training (2, 12–14). Many guidelines emphasize that inhalers should be prescribed only after patients have been trained to use them properly and have demonstrated this ability (15, 16). However, many studies showed that overall and critical error rates of inhalers in patients with asthma or COPD were high across different devices, ranging from 50 to 100% and 14–92%, respectively, indicating that the problem of proper device use was far from solved (17). Evidence suggests that there is still a need to train patients on the correct inhalation technique for the various available devices (18).

Evidence suggested that using the manufacturer's instruction sheet alone was inadequate for patients to gain correct inhalation technique (19–21). Verbal instruction demonstrated a greater efficiency than reading the manufacturer's leaflet (20, 22, 23), especially in patients using an inhaler for the first time (21). Three instruction methods based on verbal instruction (e.g., group instruction, video instruction, and personal instruction) were proved to have good efficiency, and personal instruction (verbal education plus demonstration) was more practical than group and video instruction (24). Moreover, a review from the ADMIT series on training tools for inhalation devices concluded that inhaler technique education was best delivered by verbal instructions and physical demonstration of the technique by a skilled educator, either face to face or by video (2).

An effective training model should consider the multiple factors that impact inhaler use (25). These factors can be divided into several broad categories related to the device itself, the patient, or the health care professional (12). The most common inhaler devices are dry-powder inhalers (DPI) and pressurized metered-dose inhalers (pMDI). Both are expected to deliver a precise amount of drug in the form of aerosol particles with a particle size suitable for reaching the conducting airways when used correctly. Each drug delivery system demands a certain level of physical skill, manipulation, lung capacity and/or hand-lung coordination to ensure optimal/correct inhaler use (26). Besides physical abilities, several other patient-related factors may impact inhaler use, including patients' age, adherence (e.g., unintentional factors like poor communication between health care professionals and patients and language barriers in local dialects), and patient device preference (12, 27). In addition, healthcare professionals (e.g., respiratory physicians and pharmacists) might lack the appropriate knowledge and skills to use different inhaler devices (28). Still, as educators, they played a critical role in enabling patients to acquire the initial correct inhaler technique and maintain the proper inhaler use over time (29, 30).

At present, there needs to be more literature to report qualitative and quantitative evaluation of effective training models (considering the educators, patients, and training methods) (31). This study developed a standardized training model based on verbal instruction and device demonstration by pharmacists. Its role in improving patients' ability to use inhaler devices was evaluated qualitatively and quantitatively, compared with a usual training group based on reading the manufacturer's leaflet, a common way for Chinese patients to use before inhaler operation, although it is less efficient.

## 2. Subjects and methods

### 2.1. Study design

This study used a prospective cohort design, and two evaluations were planned for the standardized training group. The first evaluation was performed immediately after the first-time or re-training process, while the second was done more than 9 months after the first evaluation. This paper reported only the preliminary results of the first evaluation.

### 2.2. Subjects

Outpatients of the Second Affiliated Hospital of Zhejiang University in Zhejiang province of eastern China were recruited for the study. The patients were numbered sequentially, and each was assigned a random number between 0 and 1 generated by the RAND function in Excel software. If the patient's random number was less than one-third, the patient was assigned to the usual training group, and if the patient's random number was more than two-thirds, the patient was assigned to the standardized training group. A qualified trainer performed the randomization, recruitment, and grouping of patients.

The inclusion criteria for participants were as follows: (a) outpatients over 14 years of age; (b) outpatients were clinically diagnosed with asthma, COPD, or asthma plus COPD according to the medical history and physical examination; (c) outpatients were prescribed with dry powder inhalers (DPI) or metered-dose inhalers (pMDI) in the first time. The following patients were excluded: (a) patients with a history of receiving verbal instruction or inhaler demonstration; (b) patients with tumor history; (c) pregnancy or lactation; (d) patients with difficulty in verbal communication because of intubation, tracheostomy, delirium, etc.; (e) patients with a history of psychiatric illness (e.g., schizophrenia, bipolar illness, and psychotic illness).

The subjects who agreed to participate in the study were asked to sign an informed consent form. The study protocol was approved by the Second Affiliated Hospital of Zhejiang University institutional committee (Protocol number: 2021-0081) and the Chinese Clinical Trial Registry (website: chictr.org.cn, registration number: ChiCTR2100043592, 23-02-2021). All study methods, including study type, study objectives, study design, inclusion or exclusion criteria, grouping or intervention, sample size, and measurement methods were performed in accordance with the relevant guidelines and regulations required by the Chinese Clinical Trial Registry.

Patient data, such as general information (age, gender, education, living area), type of respiratory diseases, type of inhaler devices, follow-up physician, and history of hospitalization, were extracted from an integrated electronic prescribing system of the hospital.

Illiteracy or elementary school (duration of education was <7 years), middle or high school (duration of education was between 7 and 12 years), and university (duration of education was more than 12 years) were included in our study.

pMDI usually uses medications such as Albuterol Sulfate and Ipratropium Bromide. The main types of DPI were turbuhaler (using drugs like Budesonide, Budesonide and Formoterol), discus (using drugs like Fluticasone and Salmeterol), and breezhaler (using Tiotropium).

The level of patient adherence was also investigated using the Medication Adherence Report Scale (MARS)-5, which consists of five general statements about non-adherent behavior (32, 33). The five statements are: “I forget to take my medicines,” “I alter the dose of my medicines,” “I stop taking my medicines for a while,” “I decide to miss out a dose, and “I take less than instructed.” These questions were answered on a 5-point Likert scale (1 = always, 2 = often, 3 = sometimes, 4 = rarely, 5 = never). The scores were summed with a possible range of 5–25. A score of 5–22 was considered non-adherent, and a score of 23–25 was considered adherent.

### 2.3. Intervention and control

#### 2.3.1. Standardized training model for inhaler use

**Figure 1:**
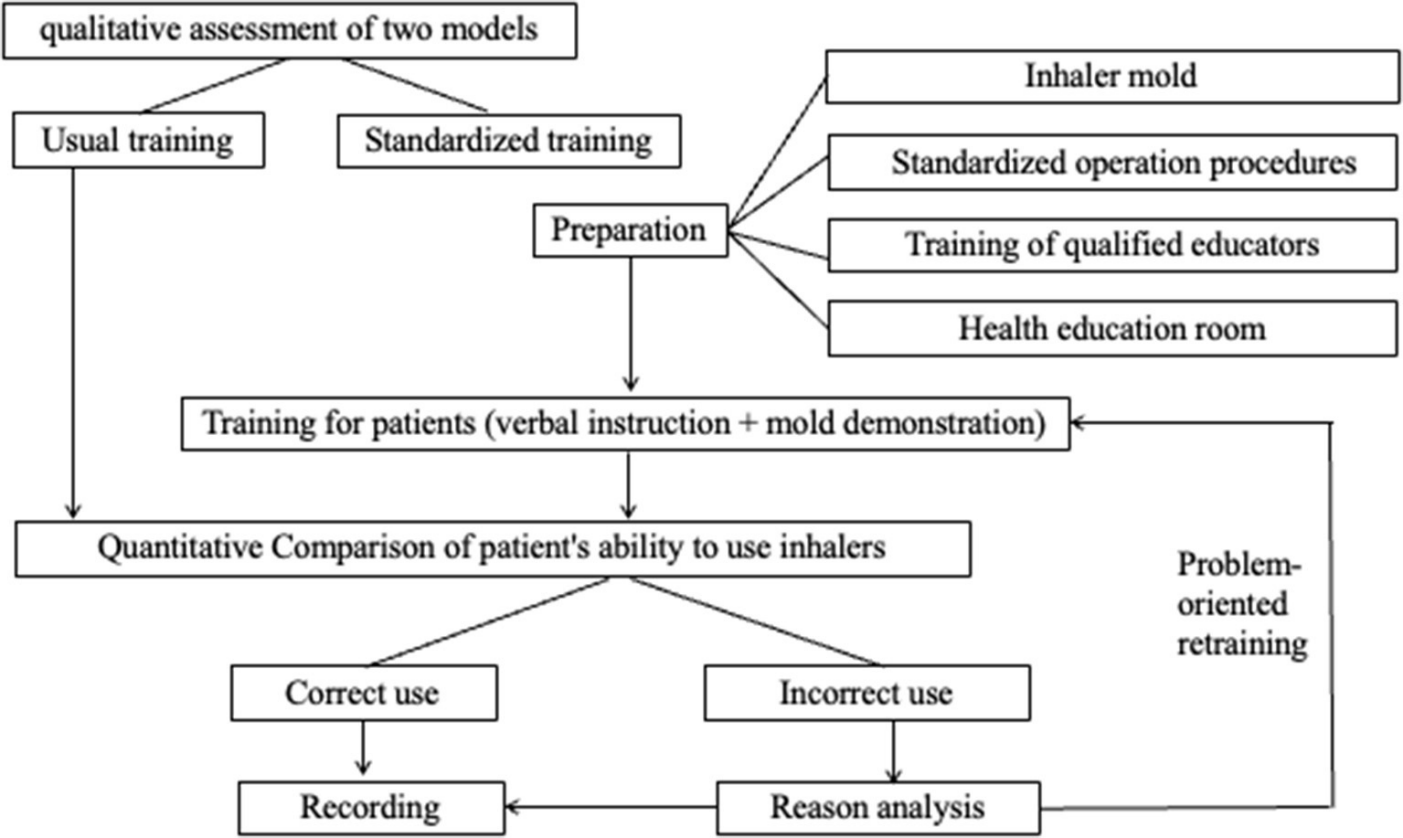
Flow chart of standardized training model.

A standardized training model was developed based on verbal instruction and mold demonstrations to help patients improve their abilities to use different types of inhaler devices properly. Figure 1 demonstrates the flow chart of the standardized training model. It was described briefly below:

1. Preparation before patient training. The following four parts need to be prepared: (a) Standard demonstration devices (inhaler molds) were provided by the manufacturer. The mold matched the inhaler prescribed by physicians; (b)The standardized operation procedure is formulated for correct use of each inhaler device based on the manufacturer's leaflet; (c) Training for qualified educators or trainers. Pharmacists from the hospital pharmacy, as participating investigators, were trained to be skilled trainers and received ongoing training; and (d) A health education room was prepared specifically for the interaction between trainers and patients.
2. Training for patients: each participant received the standardized training course based on a trainer's verbal instruction and inhaler demonstration. The training program for patients included the following knowledge and skills: (a) The importance of the correct use of inhalers for successful treatment; (b) The operation procedure of mold in the form of written instructions and pictures, including a written plan for the use of the medication; (c) The Techniques for correct use of each inhaler mold; (d) The improvement of patient adherence against unintentional factors (e.g., not understanding therapy correctly), especially in the problem-oriented re-training stage in the standardized training procedure.
3. Evaluation of patient's ability to use inhalers: After the first-time standardized training, trainers assessed participants' ability to use inhaler mold. A checklist for assessing the inhaler use ability was developed based on the “A Guide to Aerosol Delivery Devices for Respiratory Therapists 4th Edition” published by the AARC (American Association of Respiratory Care) and “Guidelines for bronchial asthma prevent and management (2020 edition)” released by the AGCTS (34). The evaluation method was used to determine the type of errors patients made and the correct use (CU). Table 1 lists the definition of operating error in the standardized operation procedures for inhaler devices, in which incorrect use (IU) included complete error (CE) and partial error (PE) (12, 25).
4. Reason analysis, re-training, and recording: If the patient was evaluated with IU, the reasons influencing the patient's ability of device use should be analyzed in terms of their skills of inhaler use, medical adherence, vulnerability (e.g., older age and low educational level), and training methodology. The evaluation conclusion and problem analysis were recorded by the trainer. Then, the re-training was performed immediately after the first training for patients evaluated with IU based on problem analysis, and the evaluation was done according to the criteria in Table 1.

**Table 1:** Definitions for operating errors of inhalers in standardized training programs.

**Type of error**	**Type of inhaler device**	**Definition**
Complete error (CE)	pMDI	• Failing to remove the cap before use • Not holding the inhaler upright • Actuation is not coordinated to breathing: actuation before breathing or actuation is too late • Failure to actuate • Failure to inhale • Failure to inhale through mouthpiece: putting the wrong end of an inhaler in the mouth; firing pMDI into the mouth but inhaling through the nose • Failure to know when the device is empty and waste remaining doses • Firing pMDI multiple times during single inhalation • Excessive use of pMDI beyond rated capacity (loss of dose count)
	DPI	• Failure to remove cap or slide cover • Blowing into the device before inhalation • Failure to inhale through the mouthpiece • Inhalation is not forceful from the start • Failure to know when the device is empty
	Turbuhaler	• Not holding the inhaler upright • Does not prepare correctly- twisting the base until it clicks or turning it back to the original position.
	Discus or Accuhaler	• Does not prepare correctly- Failure to slide lever
	Breezhaler	• Failure to insert or pierce the capsule
Partial error (PE)	pMDI	• Inadequate priming/shaking/mixing before use • Failure to breathe out before inhalation or exhaling during actuation • Too rapid inspiratory flow rate • Stop inhaling prematurely or abrupt discontinuation of inspiration as aerosol hits throat • Failure to hold the breath (< 3 s) • Failure to rinse mouth after inhalation
	DPI	• The dose is not prepared correctly-shaking during and after preparation • Failure to breathe out before inhalation • Slow and unforced inhalation • Stop inhaling prematurely, suggesting total lung capacity is not reached • Failure to hold the breath (< 3 s) • Failure to rinse mouth after inhalation • Do not control whether some powder drug rests into the capsule after inhalation (for single-dose DPI)

pMDI, pressurized metered-dose inhaler; DPI, dry powder inhaler.

#### 2.3.2. Usual training model (control) for inhaler use

This study used the usual training model as a control for the standardized training model. Usual training means that patients can use the inhaler device based on their understanding by reading the manufacturer's instructions which contain information about how to use the inhalers step by step with an illustration. It is common for patients to use inhalers in China, although it is less efficient.

### 2.4. Outcomes

#### 2.4.1. Comparative study of the two models

In order to evaluate the effect of the standardized training model on improving patients' ability to use the inhaler correctly, a comparative study was designed to compare qualitative and quantitative differences between the standardized training model and the usual training model. Multi-criteria analysis was performed to compare the qualitative differences (35). Quantitative comparisons were performed based on a statistical difference in the percentage of correct use (CU%) or percentage of incorrect use (IU%) between the two models. In addition, the changes in key factors influencing patients' ability to use inhalers were observed. Finally, the consistency of qualitative and quantitative comparisons was evaluated.

#### 2.4.2. Qualitative comparisons

Key information regarding the targeted population, attribute, trainer and assessor, interaction, validation, reliability, guidance, error correction mechanism, and operability of the two models was qualitatively analyzed based on the literature review and expert discussion. The literature databases queried were Web of Science, Pub-Med, Medline, Scopus, and related official websites. Search terms used were “inhaler,” “inhalation technique,” “Asthma,” “Chronic obstructive pulmonary disease (COPD),” “education,” “training,” or “aerosol delivery therapy.”

Multi-criteria qualitative analysis was subsequently established based on this analysis of essential information. It included the following steps: determination of evaluation indicators, assignment of indicator values and weights, expert consultation, interview with key informants, and comprehensive analysis. The evaluation indicators were determined based on the literature review and expert consultation, in which 20 experts in the field of health management, pharmacist, or respiratory physician were asked for advice on evaluating indicators in two rounds. The nine selected indicators are shown in Table 2. To assign indicator weight, 85% of the consulted experts agreed that the weight of the nine indicators should be equivalent, meaning that each indicator was equally important. Furthermore, most experts (90%) considered it appropriate to divide each indicator into low, medium, and high levels, which were assigned 1, 2, and 3 points, respectively. However, the interaction (the interactive model between the patient and trainer is better), error correction mechanism (the model with an error correction mechanism is better), and operability (the model with simple steps is convenient to use) indicators were only divided into two levels (low and high), because the medium level was difficult to define.

**Table 2:** Scoring system used for the multi-criteria analysis for training models.

**Criteria (indicators)**	**Scores (levels)**		
	**1 (Low)**	**2 (Medium)**	**3 (High)**
Targeted population (The model applies to all populations covering vulnerable populations is more useful.)	General population	General population at least including one kind of vulnerable population like children, teenagers, or the elderly	General population covering vulnerable populations like teenagers, and the elderly
Attribute	Self-education	Verbal instruction or video instruction	Verbal instruction and physical demonstration
Trainer or educator (The training model that is performed and evaluated by a qualified trainer is effective.)	None	Untrained healthcare professionals	Qualified healthcare professionals
Interaction (The interactive model between the patient and trainer is better.)	No	-	Mutual interaction between patient and trainer
Validation (The model that is validated by documents containing independent data may be more accurate.)	No	Validation by a few documents	Validation by adequate documents with independent data
Reliability (The model that has a standardized or systemic training procedure is more reliable.)	No	Simple training procedure	Standardized or systemic training procedure
Guidance (The model provides an explanatory guidance and device demonstration that help implementation.)	No	Written guidance manual	Written guidance manual and device demonstration
Error correction mechanism (The model with a error correction mechanism and retraining is better)	No	-	Corrective mechanism and retraining for operational errors
Operability (The model with simple steps is convenient to use.)	Complicated to use because steps are complex or time-consuming	-	Easy to use because steps are simple or save time

A radar diagram was drawn to directly reflect the distribution of the two methods at different levels for each evaluation indicator. The total score for each model in the nine evaluation indicators was calculated to evaluate whether each model had a comprehensive advantage.

#### 2.4.3. Quantitative comparisons

In order to observe the role of the standardized training model, the difference in CU% or IU% between the standardized training group and the usual training group was analyzed using a stratified analysis by gender, age, education level, living area, type of follow-up physician, history of hospitalization, inhaler type, DPI subtype, and the MARS-5 scores of patient's adherence. In this study, the first-time training and re-training effects of the standardized procedure were investigated to provide a basis for a follow-up study in the future.

In addition, the changes in key factors influencing the ability to use inhalers were investigated after standardized training compared with usual training.

### 2.5. Sample size

The sample sizes of the two groups were calculated by the Z-test of PASS software (Tests for two proportions) (37). The average percentages of CU for standardized training and usual training were at least 70% and 30%, respectively, based on the literature review and pre-investigation. If α = 0.05, β = 0.1, and the dropout ratio is 20%, if the sample size of the two groups is equal, at least 39 usual training patients and 39 standardized training patients should be included. That is, the minimum sample size for this study is 78.

### 2.6. Statistical analysis

The Chi-square analysis was used to analyze the CU% or IU% of the two models and the association between CU% (or IU%) and influencing factors. The binary logistics regression analysis was used to analyze the OR and 95% CI of factors influencing inhaler use ability. *P*-value < 0.05 was considered statistically different.

## 3. Results

### 3.1. Characterization of included patients

A total of 431 outpatients were recruited in the study, i.e., a standardized training group with 280 patients and a usual training group with 151 patients. There were no significant differences in gender, age, educational level, occupational status, living area, MARS-5 scores of adherence, type of respiratory diseases, combined use of MDI and DPI between the two groups (*P* > 0.05, Table 3). The average age of patients was 52.6 ± 0.78 years. Among all subjects, 53.6% were female, 45.7% were illiterate or have an elementary school education, 30.9% were farmers or workers, 23.4% lived in rural areas. Patients suffering from asthma, COPD and asthma+COPD accounted for 64.5%, 26.2%, and 9.3%, respectively. 12.3% of patients used MDI combined with DPI or used more than two different DPI types simultaneously.

**Table 3:** General information of subjects and the inhaler device used in the two groups.

**Characteristic**	**Usual training (*n*, %) (*n* = 151)**	**Standardized training (*n*, %) (*n* = 280)**	**Total (*n*, %) (*n* = 431)**	***P***
**Gender**				0.840
Male	69 (45.7)	131 (46.8)	200 (46.4)	
Female	82 (54.3)	149 (53.2)	231 (53.6)	
Age, mean in years (SEM)	51.8 (1.3)	53.1 (0.96)	52.6 (0.78)	0.449
**Education level**				0.264
Illiteracy or elementary school	62 (41.1)	135 (48.2)	197 (45.7)	
Middle or High school	40 (26.5)	73 (26.1)	113 (26.2)	
University	49 (32.5)	72 (25.7)	121 (28.1)	
**Occupational status**				0.534
Farmers and workers	43 (28.5)	90 (32.1)	133 (30.9)	
Officers	65 (36)	102 (36.4)	167 (38.7)	
Businessman	19 (12.6)	44 (15.7)	63 (14.6)	
Others	24 (15.9)	44 (15.7)	68 (15.8)	
Living area				0.233
Urban center	121 (80.1)	209 (74.6)	330 (76.6)	
Rural area	30 (19.9)	71 (25.4)	101 (23.4)	
**MARS-5 scores**				0.248
< 23	90 (59.6)	184 (65.7)	274 (63.6)	
23–25	61 (40.4)	96 (34.3)	157 (36.4)	
**Type of respiratory diseases**				0.249
Asthma	96 (63.6)	182 (65)	278 (64.5)	
COPD	38 (25.2)	75 (26.8)	113 (26.2)	
Asthma+ COPD	17 (11.3)	23 (8.2)	40 (9.3)	
**Combined use of MDI and DPI**				0.818
MDI+DPI+DPI	1 (0.7)	2 (0.7)	3 (0.7)	
MDI+DPI	1 (0.7)	5 (1.8)	6 (1.4)	
DPI+DPI	17 (11.3)	27 (9.6)	44 (10.2)	
No	132 (87.4)	246 (87.9)	378 (87.7)	
**Inhaler type**				1.000
pMDI	6 (3.5)	12 (3.8)	18 (3.7)	
DPI	166 (96.5)	305 (96.2)	471 (96.3)	
Turbuhaler	78 (45.3)	154 (48.6)	232 (49.3)	
Discus	53 (30.8)	99 (31.2)	152 (32.3)	
Breezhaler	35 (20.3)	52 (16.4)	87 (18.4)	

COPD, chronic obstructive pulmonary disease; pMDI, pressurized metered dose inhalers; DPI, dry powder inhalers.

### 3.2. Characterization of included training devices

A total of 489 training devices in two groups were included in the study, i.e., the standardized training group with 317 devices and the usual training group with 172 devices. The DPI and pMDI inhaler device use percentages were 96.3% and 3.7%, respectively. The types of DPI were turbuhaler (49.3%), discus (32.3%), and breezhaler (18.4%) (Table 3).

### 3.3. Qualitative differences between the two models

**Figure 2:**
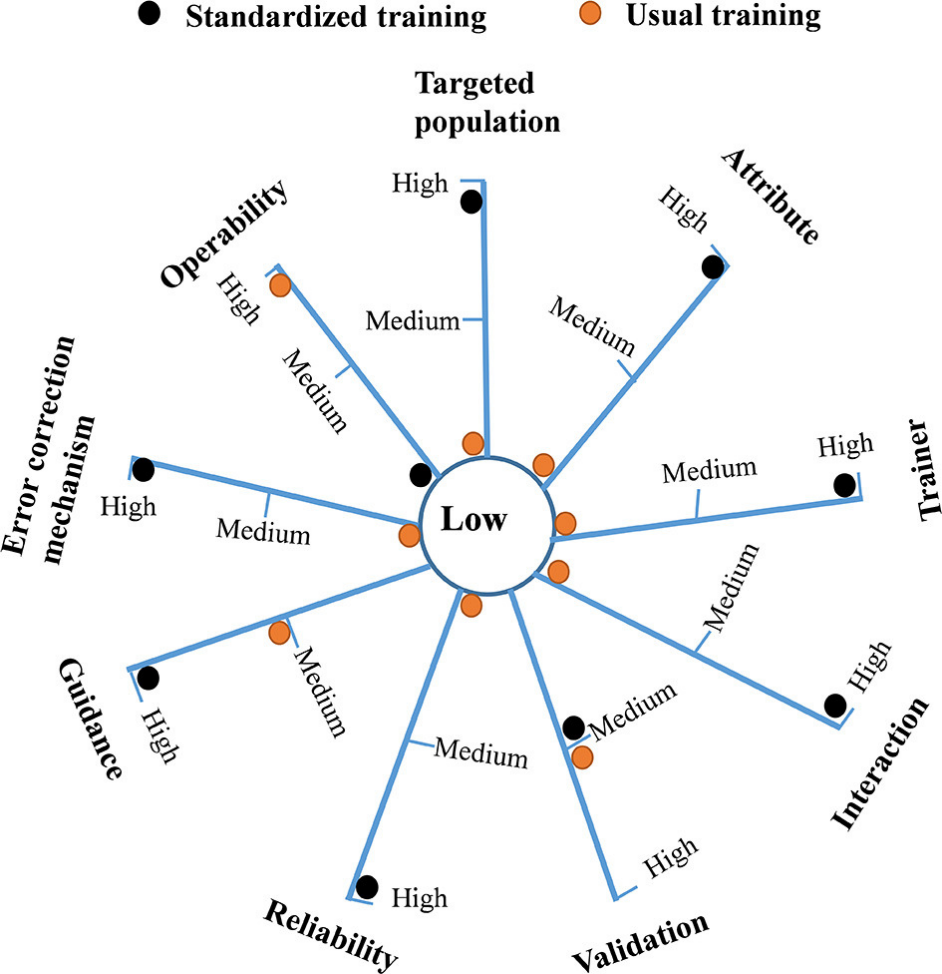
A radar diagram reflecting the distribution of the usual training model and standardized training model at different levels for each qualitative indicator. The total score of the standardized training model and usual training model were 24 and 13, respectively.

The radar diagram (Figure 2) directly reflects the distribution of the two methods at different levels for each qualitative indicator. The multi-criteria analysis shows that the total score of the standardized training model and usual training model were 24 and 13, respectively. The standardized training model had significant advantages over the usual model in qualitative indicators of the targeted population (3 vs. 1), attribute (3 vs. 1), trainer (3 vs. 1), interaction (3 vs. 1), reliability (3 vs. 1), guidance (3 vs. 2), and error correction mechanism (3 vs.1). The two models had a similar score in the validation indicator (2 vs. 2). Only the operability indicator of the usual training model was better than that of the standardized training model (3 vs. 1).

### 3.4. Quantitative differences between the two models

Table 4 shows quantitative differences in the inhaler device use between the two models at different subgroups of each factor. A total of 489 training devices for two groups were included. The CU% of each subgroup of each factor (e.g., gender, age, educational level, living area, follow-up physician, history of hospitalization, inhaler type, and MARS-5 scores of adherence) in the standardized training group after first-time training was significantly higher than that of the usual training model (*P* < 0.05). The subgroups with the most significant increase of CU% in the standardized training group after first-time training as compared with the usual training group were: living in the rural area (increasing by 52.4%), illiteracy or elementary school (52.4%), ≥60 years old (increasing by 45.3%), and the score of adherence <23 (increasing by 41.5%). Correspondingly, the average percentage of incorrect use (IU%) in the standardized training group after first-time training (28.4%) was significantly lower than that of the usual training group (64.5%). Moreover, the average percentage of complete error (CE%) in IU% in the standardized training group after first-time training was 1.6%, which was significantly lower than that (30.8%) of the usual training group (*P* < 0.05). The average percentage of partial error (PE%) in IU% in the standardized training group after first-time training was 26.8%, which was significantly lower than that (33.7%) of the usual training group (*P* < 0.05). In terms of DPI subtypes, the CU% of Turbuhaler, Discus, and Breezhaler in the usual training group were 38.5%, 34.0%, and 34.3%, respectively, which were significantly lower than those (64.3%, 74.7%, and 92.3%, respectively) in the standardized training group after first-time training (*P* < 0.05).

**Table 4:** Quantitative differences in inhaler use ability between the two models using a stratified analysis.

**Factor**	**Usual training group (*****n*** = **172)**			**Standardized training group**								
				**After first-time training (*****n*** = **317)**				**Re-training No. (*****n*** = **90)**	**After re-training (*****n*** = **317)**			
	**CU (** ***n*** **, %)**	**IU (** ***n*** **, %)**	***P***	**CU (n, %)**	**IU (** ***n*** **, %)**	***P***	**D1 (%)**		**CU (** ***n*** **, %)**	**IU (** ***n*** **, %)**	***P***	**D2 (%)**
**Gender**			0.715			0.075					0.075	
Male	29 (34.1)	56 (65.9)		106 (67.1)	52 (32.9)		33^a^	52	116 (73.4)	42 (26.6)		6.3
Female	32 (36.8)	55 (63.2)		121 (76.1)	38 (23.9)		39.3^a^	38	130 (81.8)	29 (18.2)		5.7
**Age (years)**			0.015			0.674					0.234	
18-60	43 (43.0)	57 (57.0)		137 (72.5)	52 (27.5)		29.5^a^	52	151 (79.9)	38 (20.1)		7.4
≥60	18 (25.0)	54 (75.0)		90 (70.3)	38 (29.7)		45.3^a^	38	95(74.2)	33(25.8)		3.9
**Education level**			0.004			0.276					0.761	
Illiteracy or elementary school	16 (21.6)	58 (78.4)		114 (74.0)	40 (26.0)		52.4^a^	40	120(77.9)	34 (22.1)		3.9
Middle or High school	21 (44.7)	26 (55.3)		62 (73.8)	22 (26.2)		29.1^a^	22	63(75)	21(25)		1.2
University	24 (47.1)	27 (52.9)		51 (64.6)	28 (35.4)		17.5^a^	28	63 (79.7)	16 (20.3)		15.1
**Living area**			0.221			0.104					0.113	
Urban center	52 (37.7)	86 (62.3)		167 (69.3)	74 (30.7)		31.6^a^	74	182 (75.5)	59 (24.5)		6.2
Rural area	9 (26.5)	25 (73.5)		60 (78.9)	16 (21.1)		52.4^a^	16	64 (84.2)	12 (15.8)		5.3
**Type of follow-up physician**			0.202			0.379					0.484	
Chest disease specialist	53 (34.0)	103 (66.0)		211 (72.3)	81 (27.7)		38.3^a^	81	228 (78.1)	64 (21.9)		5.8
Internal medicine specialist	8 (50.0)	8 (50.0)		16 (64.0)	9 (36.0)		14.0^a^	9	18 (72)	7(28)		8
**History of hospitalization**			0.437			0.845					0.765	
Yes	14 (41.2)	20 (58.8)		27 (73.0)	10 (27.0)		31.8^a^	10	28 (75.7)	9 (24.3)		2.7
No	47 (34.1)	91 (65.9)		200 (71.4)	80 (28.6)		37.3^a^	80	218 (77.9)	62 (22.1)		6.5
**Inhaler type**			0.425			0.091					0.149	
pMDI	1 (16.7)	5 (83.3)		6 (50.0)	6 (50.0)		33.3^a^	6	7 (58.3)	5 (41.7)		8.3
DPI	60 (36.1)	106 (63.9)		221 (72.5)	84 (27.5)		36.4^a^	84	239 (78.4)	66 (21.6)		5.9
**DPI subtype**			0.842			0.000					0.002	
Turbuhaler	30 (38.5)	48 (61.5)		99 (64.3)	55 (35.7)		25.8^a^	55	113 (73.4)	41 (26.6)		9.1
Discus	18 (34.0)	35 (66.0)		74 (74.7)	25 (25.3)		40.7^a^	25	77 (77.8)	22 (22.2)		3.1
Breezhaler	12 (34.3)	23 (65.7)		48 (92.3)	4 (7.7)		26.6^a^	4	49 (94.2)	3 (5.8)		1.9
**Combined use of MDI and DPI**			0.553			0.220					0.222	
Yes	0(0)	3 (100)		8 (57.1)	6 (42.9)			6	9 (64.3)	5 (35.7)		
No	61 (36.1)	108 (63.9)		219 (72.3)	84 (27.7)			84	237 (78.2)	66 (21.8)		
**Simultaneous use of DPI devices**			0.489			0.000					0.000	
Yes	11 (30.6)	25 (69.4)		53 (94.6)	3 (5.4)			3	54 (96.4)	2 (3.6)		
No	50 (36.8)	86 (63.2)		174 (66.7)	87 (33.3)			87	192 (73.6)	69 (26.4)		
**MARS-5 Score of adherence**			0.001			0.014					0.019	
< 23	25 (25.3)	74 (74.7)		133 (66.8)	66 (33.2)		41.5^a^	66	146(73.4)	53(26.6)		6.6
23-25	36 (49.3)	37 (50.7)		94 (79.7)	24 (20.3)		30.4^a^	24	100(84.7)	18(15.3)		5
**Average**	61 (35.5)	111 (64.5)		227 (71.6)	90 (28.4)		36.1^a^		246(77.6)	71(22.4)		6
CE% in IU%	-	53 (30.8)		-	5 (1.6)		29.3^a^			0(0)		1.5
PE% in IU%	-	58 (33.7)		-	85 (26.8)		6.9^a^			71(22.4)		4.4

CU,correct use; IU, incorrect use, including complete error (CE) or partial error (PE);

D1, difference of CU% or IU% between usual training group and first-time standardized training group; D2, difference of CU% or IU% between standardized training group after first-time training and after retraining training;

a *P* < 0.05, significant differences in CU% or IU% between usual training group and standardized training group after first-time training.

After the first-time training, 90 devices were evaluated with IU and accepted re-training. The average CU% of the usual training and standardized groups after re-training were 35.5% and 77.6%, respectively, increasing by 44.1%. After an increase of 36.1% in CU% in the first standardized training session compared with usual training, the CU% of the re-training session increased by 6% again. However, no significant difference was observed between the two training sessions. After re-training, the CE% in IU% was zero, and the PE% in IU% remained 22.4%, decreased by 4.4%.

### 3.5. Key factors influencing patients' ability to use inhaler devices

Table 4 shows that in the usual training group, IU% among patients with age ≥60 years old was significantly higher than that of those aged 18–60 years old (*P* < 0.05); IU% of the patient with illiteracy or elementary level was significantly higher than that of the patient with middle or high school or college education (*P* < 0.05); IU% of the patient with MARS-5 score of adherence score <23 was significantly higher than that of the patient with adherence score of 23–25 (*P* < 0.05). Although the CU% of DPI in the usual training group or the standardized training group was higher than that of pMDI, the difference was not statistically significant (*P* > 0.05).

In the standardized training group after first-time training or re-training, the IU% of the patients with adherence scores <23 was significantly higher than that of the patients with adherence scores of 23–25 (*P* < 0.05). IU% of Turbuhaler or Discus subtype was higher than that of Breezhaler subtype (*P* < 0.01); The CU% of the patients using DPI devices simultaneously was higher than that using one DPI (*P* < 0.01).

Logistic regression analysis in Table 5 illustrates the key factors influencing the ability to use inhaler devices for the two models. In the usual training group, the ORs (95%CI) of age, educational level, and patient adherence were 2.263 (1.165–4.398), 0.556 (0.379–0.815), and 0.347 (0.182–0.662), respectively. In the standardized training group, after the first-time training or re-training, age and educational level were not the key factors influencing the ability to use inhaler devices (*P* > 0.05). Better patient adherence with MARS-5 scores 23–25 was still the key factor impacting patients' ability to use inhalers (OR = 0.515, 95%CI: 0.301–0.880) as compared with patients' adherence with a MARS-5 score of <23. Simultaneous use of DPI devices was also influencing factors.

**Table 5:** Logistic regression analysis of key factors influencing the ability to use inhalers for the two models.

**Factors**	**Usual training**			**Standardized training**					
				**First-time training**			**Re-training**		
	**OR**	**95% CI**	***P***	**OR**	**95% CI**	***P***	**OR**	**95% CI**	***P***
Age	2.263	1.165–4.398	0.016	1.112	0.678–1.826	0.674	1.380	0.811–2.351	0.235
Education level	0.556	0.379–0.815	0.003	1.233	0.920–1.654	0.161	0.968	0.702–1.336	0.844
Patient's adherence	0.347	0.182–0.662	0.001	0.515	0.301–0.880	0.015	0.496	0.274–0.896	0.02
DPI subtype	1.185	0.823–1.706	0.362	0.682	0.500–0.931	0.016	0.759	0.546–1.056	0.101
Simultaneous use of DPI devices	1.321	0.600–2.912	0.489	0.113	0.034–0.373	0.000	0.103	0.024–0.434	0.002

Age (years), 18–60, ≥60; Education level, illiteracy or elementary school, middle or High school, university; Patient's adherence, MARS-5 score <23, 23–25; DPI subtype, Turbuhaler, Discus, Breezhaler; Simultaneous use of DPI devices: no, yes. In usual training group, older age is risk factor for incorrect use, and high education level and better adherence are preventive factors incorrect use; in standardized training (including retraining), better adherence, simultaneous use of DPI devices are preventive factors for incorrect use.

Logistic regression analysis for all participants in Table 6 further illustrates that after being adjusted by education level and DPI subtype, the ORs value of the training group (standardized training vs. usual training) was 0.174 (95%CI: 0.113–0.268, *P* < 0.01), and the ORs for simultaneous use of DPI devices (yes vs. no) was 0.346 (95%CI: 0.192–0.626, *P* < 0.01), and the ORs for patients' adherence (MARS-5 score 23–25 vs. <23) and age (>60 vs. 18–60) was 0.4 (95%CI: 0.260–0.617, *P* < 0.01) and 2.625 (95%CI: 1.566–4.400, *P* < 0.01), respectively.

**Table 6:** Logistic regression analysis of key factors influencing inhaler use ability in all subjects (*n* = 489).

**Factors**	**OR**	**95% CI**	***P***
Training group	0.174	0.113–0.268	0.000
Age	2.625	1.566–4.400	0.000
Education level	0.988	0.752–1.298	0.928
Patient's adherence	0.400	0.260–0.617	0.000
DPI subtype	0.791	0.613–1.020	0.071
Simultaneous use of DPI devices	0.346	0.192–0.626	0.000

Training group, usual training; standardized training (including retraining); Age (years), 18–0, ≥60; Education level, illiteracy or elementary school, middle or High school, university; Patient's adherence: MARS-5 score <23, 23–25; DPI subtype, Turbuhaler, Discus, Breezhaler; Simultaneous use of DPI devices, no, yes. Older age is risk factor for incorrect use, and standardized training (including retraining), better adherence and simultaneous use of DPI devices are preventive factors for incorrect use.

## 4. Discussion

This study attempts to evaluate the efficacy of a standardized training model by pharmacists based on verbal instructions and device demonstration in enhancing patients' inhalation ability using qualitative and quantitative analysis.

From the perspective of qualitative analysis, the radar diagram demonstrates that the standardized training model had significant advantages in indicators of the targeted population, attribute, trainer, interaction, reliability, guidance, and error correction mechanism. The standardized training model focused on the general population that covered vulnerable people like teenagers and the elderly. Since this hospital was not a children's hospital, the study sample did not involve children. As a standard method, the usual training model is unsuitable for elderly patients and adolescents with relatively poor comprehension of written materials. The methodological attributes of the standardized training model were verbal instruction and physical demonstration, which are more effective than the usual training model, as many studies have shown that verbal instruction or physical demonstration was more efficient than manufacturer's leaflet (21, 24). The standardized training model of this study was designed to specifically provide continuous training to trainers or educators until their inhaler competency was qualified since healthcare professionals typically lack the appropriate knowledge and skills in using different inhaler devices (29). Evidence suggested that community pharmacists receiving single-session training could significantly improve patients' inhaler techniques and asthma outcomes by providing inhaler education interventions (38). In the standardized train model, the knowledge and abilities that a qualified trainer should possess were specified in detail, including familiarity with training procedures, basic knowledge of respiratory medicines and mechanisms of action, the importance of correct use of inhalers, common errors of inhaler use and corresponding interventions, good skills of teaching, demonstration, evaluation, and intervention. This standardized training program also focused on problem-oriented re-training, with the primary goal of eliminating complete errors and improving partial errors of inhaler use. In this study, after re-training, the CE% in IU% was zero, the PE% in IU% remained 22.4%, decreasing by 4.4%.

In addition, the standardized training model was designed to have a good interaction between the patient and the trainer through face-to-face, provide explanatory guidance and device demonstration, and have an error correction mechanism based on problem-oriented re-training for patients, which might lead to higher training efficiency. Moreover, the standardized training model might exhibit better reliability than usual training since the model had a standardized or systemic training procedure (Figure 1). There is a need to train patients more effectively in following a proper sequence of inhalation steps to ensure maximum delivery of an inhaled drug to receptor sites in the lungs (2). The correct sequence of inhalation steps should be solidified or standardized for ease of use and generalization. The two models scored similarly on the validation indicator because more independent data was still required to validate the two models. Only the score of the operability indicator for the standardized training model was lower than that of the usual training model because of the complexity and time-consuming of the standardized training method. If a patient was evaluated for incorrect use, factors influencing the patient's inhaler use would be analyzed, and re-training continued, then the process was recorded. The whole process would take 3–7 min. The multi-criteria analysis showed that the total score of the standardized training model was 24, which was greater than that (13) of the usual training model. This result reflected that the standardized training model had comprehensive advantages in key qualitative indicators.

The above methodological advantages from qualitative analysis were supported by the quantitative analysis in this study. The average CU% of the standardized training group was 77.6%, which was greater than that (35.5%) of the usual training group (*P* < 0.05). The stratified analysis demonstrated that CU% in each factor subgroup for the standardized training model was significantly higher than the usual training model (*P* < 0.05). The increase of CU% in the standardized training group was particularly significant for subgroups such as those living in a rural area, illiterate or elementary school level, elderly patients, and poor medication adherence. Correspondingly, the average PE% (partial error) or CE% (complete error) in IU% (incorrect use) in the standardized training group was significantly lower than the usual training group (*P* < 0.05). The CU% of DPI (e.g., Turbuhaler, Discus, and Breezhaler) in the usual training group was <40%, and it increased significantly to 72.5% in the standardized training group after first-time training, then reached 78.4% after the re-training mainly through eliminating the complete error. These quantitative differences indicated that the training efficiency of the standardized training was much higher than that of usual training, and the newly established standardized training programs improved patients' ability to use inhalers correctly. The reason for efficiency gains might be related to the interactive and error-correcting training methodology, based on a standardized training process using face-to-face verbal instruction and inhaler mold demonstration by qualified trainers. These results were supported by previous studies. Ronmark et al. carried out a randomized multicenter parallel-group trial to compare correct use and acceptability of Diskus, Turbuhaler, and Easyhaler powder inhalers among 326 patients. They found that when the subjects were only asked to read the manufacturer's leaflet, only half of the subjects used the DPI devices correctly, while video education lasting 4 weeks based on verbal instruction could increase CU% to 80–90% (19). Serra-Batlles et al. reported that the percentage of correct handling maneuvers and the percentage of patients who achieved correct maneuvers increased significantly after the first verbal instruction compared with reading the manufacturer's leaflet (21). Bosnic-Anticevich et al. investigated the effect of two educational interventions on improving the MDI technique delivered in community pharmacy. They concluded that adding a physical demonstration was more effective in enhancing the pMDI technique than written and verbal instructions alone (39). ADMIT also believes that a better patient training tool for inhalation devices would combine verbal instruction with physical demonstration (2).These findings suggested that usual training based on the manufacturer's instruction sheet alone was not enough to improve the patient's inhalation technique. A combination of verbal instruction (with written materials) and physical demonstration exhibited better training efficiency. Many studies further suggested that repeating the combined education over time could increase the proportion of patients returning to follow-up visits who maintain the correct technique (38, 39).

An effective training methodology must overcome the influence of factors affecting patients' inhalation ability. Old age, low education level, and poor adherence were crucial factors affecting usual training patients' ability to use inhalers correctly. The standardized training could significantly improve the CU% of inhalers in vulnerable groups such as elderly patients and patients with low education levels. Elderly patients are less able to use inhalers properly since age can change anatomic and physiologic factors such as airway size, respiratory rate, and lung volume (40, 41). Often, the very young and the elderly experience physical difficulties when using an inhaler device (42). The low educational level of the patient might affect their cognitive ability to understand how and when to use a device and medication, as well as their physical ability and coordination in using an aerosol generator (36, 43). This study showed that the two influencing factors had been significantly addressed after this standardized training. Aydemir reported that before a face-to-face training based on verbal instruction, the patients with no education had a higher percentage of incorrect use than patients with formal education and that the difference disappeared after the training. However, older patients remained unable to use it correctly despite training (25). The age-related differences did not disappear because there was no inhaler demonstration in the training method compared with our study.

In terms of the influence of inhaler types on inhaler competence, the CU% of DPI in the usual training group or the standardized training group was higher than that of pMDI, although the difference was not statistically significant. As for DPI subtypes, there was no significant difference of CU% in usual training patients; after standardized training, the CU% of patients using Breezhaler was significantly higher than Turbuhaler or Discus. The reason for the difference in competence for different inhaler types might be that each of the different drug delivery systems demands a certain level of physical skill, manipulation, dexterity, hand strength, lung capacity, and/or hand-lung co-ordination to ensure optimal/correct inhaler use (12). Breezhaler type in this study required relatively simple operating steps and inhalation techniques for patients. Accordingly, to manage the increased risk of inhaler errors when prescribing inhalers, it is crucial to consider the individual capabilities of the patient. Table 1 describes examples of common operating errors (complete and partial errors) for each inhaler device. After standardized training, although CU% of each inhaler was improved significantly and the complete error disappeared, more than 20% of patients still had partial errors in using inhalers, such as failure to rinse mouth after inhalation, failure to hold breath, failure to breathe out before inhalation, too fast inspiratory flow rate for pMDI, and slow and unforced inhalation for DPI.

After the standardized re-training, CU% of patients with adherence scores of <23 and 23–25 were 73.4% and 84.7%, respectively, which were significantly improved than those in usual training patients, and the CU% (84.7%) of high adherence score was second only to Breezhaler DPI type (94.2%) in all subgroups. This result suggested that standardized training was more effective in improving CU% of inhalers in patients with better medication adherence because these patients were able to master breathing techniques more quickly after being instructed by the trainer. Adherence refers to a patient's choice to follow prescribed therapy. Lack of adherence can lead to poor health outcomes and increased healthcare costs, and both long- and short-term adherence may be influenced by patient and social factors (44, 45). In order to optimize disease management, patients must have the correct inhaler technique at the outset of treatment and as they continue to use their inhaler over time (38). However, studies indicate that 40%−60% of patients with asthma were non-adherent to their medication (46, 47). In this study, 63.6% of patients were non-adherent (Table 3). Failure to adhere to prescribed inhalers is categorized as “unintentional” or “intentional.” Unintentional factors usually included misunderstanding about prescribed drug regimens, such as poor communication between healthcare providers and patients, and language barriers. Intentional factors refer to understanding therapy but not adhering correctly to patient beliefs (40). The interactive standardized training programs in this study were designed to address these unintentional factors like poor communication between pharmacists and patients and language barriers in local dialects. This result was supported by the AARC, which notes that an example of unintentional non-adherence is an incorrect aerosol device technique resulting from a misunderstanding of the prescribed drug regimen, which can be corrected through patient training (31). Demonstrating operating steps could also help overcome language barriers (48). However, these intentional factors could not be addressed in this study due to the lack of corresponding intervention modules in the standardized training program. Logistic regression analysis for all participants in Table 6 further illustrates that after being adjusted by education level and DPI subtype, the standardized training model was a protective factor for patient's inhaler ability compared with the usual training, and non-adherence, old age and simultaneous use of DPI were still the main risk factors affecting patients' ability to use inhalers correctly. These findings highlighted the importance of selecting suitable training methods to address the impact of age and poor medication adherence.

Based on the above findings, the following conclusions could be drawn: (1) Compared with the usual training model, the standardized training model had comprehensive advantages in methodology; 2 Compared with usual training, standardized training could significantly improve patients' ability to use inhalers correctly, although about 22% of patients still had partial errors in using inhalers; (3) Old age and poor adherence were the main risk factors affecting patients' inhaler use ability, and a suitable training method based on verbal instruction and physical demonstration by a skilled trainer was a protective factor for correct use of inhalers in patients with asthma or COPD; (4) The framework of qualitative and quantitative comparisons could be used to evaluate different training models. More samples and well-designed population studies are needed to verify the effectiveness of the standardized training model and further improve the training methodology.

There are several limitations to be noted in this study: (a) Training tools or devices were not used in this study. The training device may allow patients to correctly and quickly master the technique and enable trainers to objectively evaluate the training efficiency by measuring patients' objective metrics like inspiratory flow and lung capacity (2); (b) The evaluation data from the follow-up required further analysis since the inhaler technique deteriorates again after education (12); (c) Failure to address the intentional factors of non-adherence was another shortcoming (31). Strategies to address this issue, such as patient-centered care and the use of motivational tools, should be incorporated into standardized training programs in future study.

## Data availability statement

The raw data supporting the conclusions of this article will be made available by the authors, without undue reservation.

## Ethics statement

The studies involving human participants were reviewed and approved by the Second Affiliated Hospital of Zhejiang University institutional committee (Protocol number: 2021-0081). Written informed consent to participate in this study was provided by the participants' legal guardian/next of kin.

## Author contributions

YH: conceptualization, methodology, data curation, and writing. FM: project administration and supervision. YD: formal analysis and data curation. CC and XZ: investigation and data collection. HD: writing-reviewing and editing. All authors gave final approval of the version to be published.
